# A proposed feasible classification of common bile duct duplications based on a newly described variant and review of existing literature

**DOI:** 10.1186/s12887-022-03708-1

**Published:** 2022-11-08

**Authors:** Huixue Sheng, Guiling Chen, Ming Yang, Hongmei Guan

**Affiliations:** Department of Radiology, Children’s Hospital of Nanjing Medical University, No.72 Guangzhou Road, Gulou District, Nanjing City, 210008 Jiangsu Province China

**Keywords:** Duplication of the common bile duct, Duplicated common bile duct, Double common bile duct, Congenital biliary dilatation

## Abstract

**Background:**

Duplication of the common bile duct (CBD) is extremely rare among the anatomical variations in the biliary tract system, which presents a septum within the CBD or an accessory CBD. In our study, we report a rare case of duplication of the common bile duct combined congenital biliary dilatation.we present a rare case of a septum in the dilated biliary tract.

**Case presentation:**

We reported a 5-year-old Asian girl who had history of repeated abdominal pain for 4 days and aggravated for 1 day. Magnetic resonance cholangiopancreatography (MRCP) examination revealed duplicated common bile duct (DCBD) malformation with congenital biliary dilatation and distal cholelithiasis. The patient underwent choledochal cyst resection and biliary tract reconstruction and abdominal cavity irrigation and drainage under general anesthesia. A septum was found within the common bile duct during the operation. The septum divided the extrahepatic bile duct into two parts connected to the left and right hepatic ducts respectively and the gallbladder is attached to the repeated right bile duct which was not previously reported in the literature.

**Conclusions:**

We complement and adjust the classification of common bile duct duplication by reviewing the literature.

## Background

The duplication of the common bile duct (DCBD) presents a septum within the common bile duct or an accessory common bile duct [1].It is important to identify this anomaly and its classification in clinical practice, as it can lead to complications and increase the risk of bile duct injury during procedures [2, 3]. In this case report, we present a rare case of a septum in the dilated biliary tract. The septum divided the extrahepatic bile duct into two parts, which were connected to the left and the right hepatic duct respectively, which has not been reported previously. The morphology of reported cases of DCBD varies greatly, and a comprehensive classification system is needed to cover newly discovered variants.

## Case presentation

**Fig. 1:**
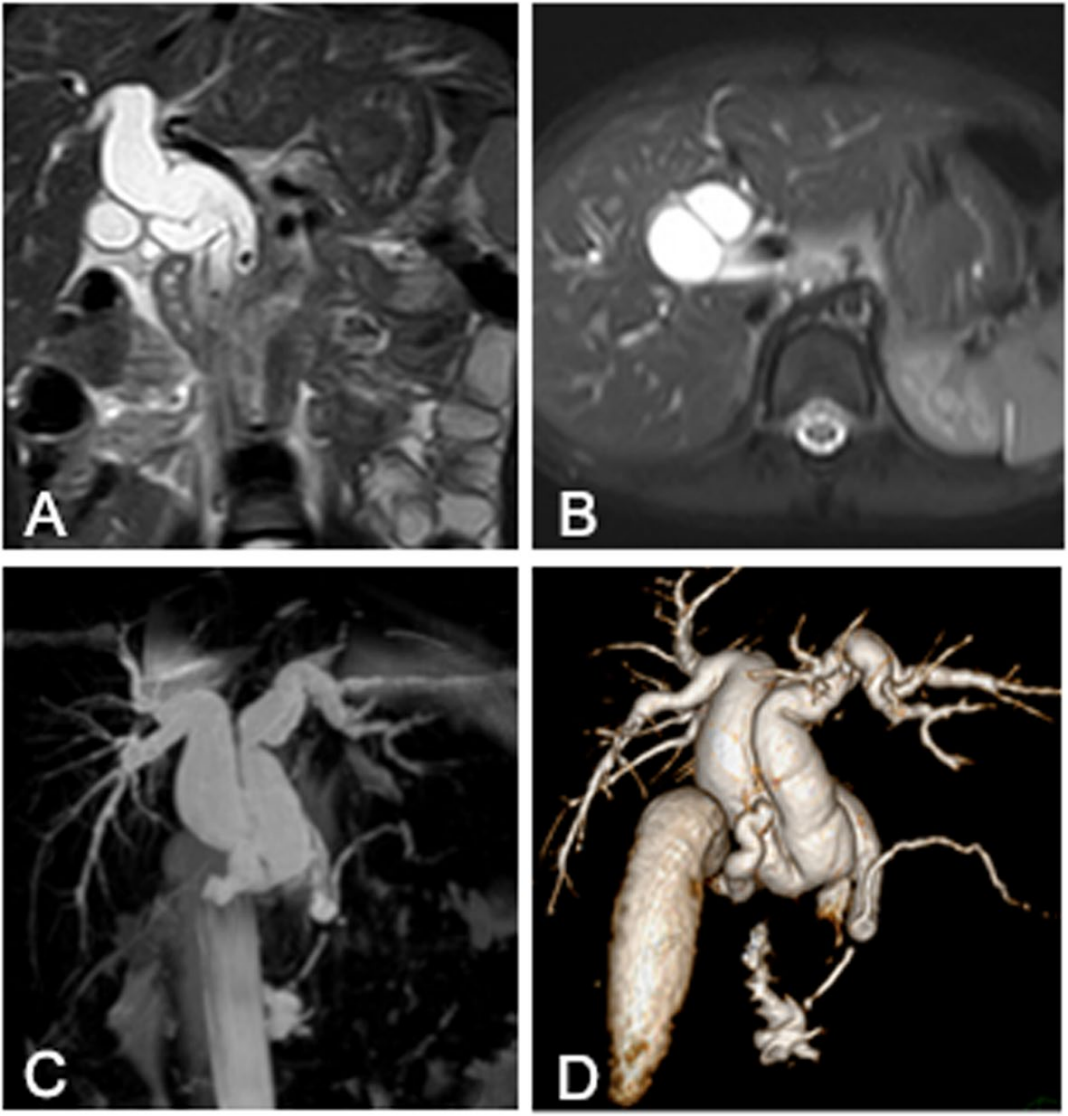
Preoperative MRCP showed the double common bile duct. **A** and **B** coronal and axial magnetic resonance T2-weighted image showed the low signal septum in the dilated common bile duct which divided the extrahepatic bile duct into two parts, which connected to the left and the right hepatic duct respectively. **C** and **D** coronal maximum intensity projection magnetic resonance image and volume rendering image showed the dilated and duplicated common bile duct

**Fig. 2:**
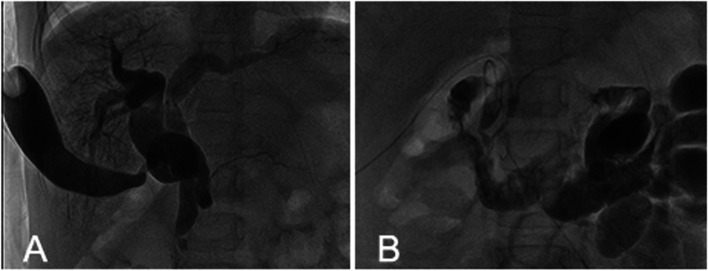
**A** IOC showed the dilated double duct and the gallbladder was connected to the duplicated bile duct. **B** IOC after operation showed that the morphology and drainage of common bile duct became normal, and large amounts of contrast medium entered into the duodenum and jejunum

The patient is a 5-year-old Asian girl who had history of repeated abdominal pain for 4 days and aggravated for 1 day. Abdominal ultrasonography suggested dilatation of common bile duct with cholangitis, cholecystitis and abdominal effusion. Magnetic resonance cholangiopancreatography (MRCP) showed a common bile duct diaphragm which meant common bile duct duplication, with congenital biliary dilatation and cholelithiasis (Fig. 1). Symptomatic treatment including fasting, anti-infection, spasmolysis, and fluid rehydration was given. The temperature of the child was stable, and there were no obvious contraindications for surgery before operation. The patient underwent cholecystectomy and biliary tract plasty and Roux-en-y common bile duct jejunostomy and abdominal cavity irrigation and drainage under general anesthesia. Intraoperative cholangiography showed both distal openings into the duodenum and converged with the pancreatic duct (Fig. 2 A). The dilated sac wall of the common bile duct was partially removed, and the septum between the left and right hepatic ducts was excised and sutured to form a regular common bile duct. Intraoperative cholangiography(IOC) after repair showed that the morphology and drainage of common bile duct was normal, and large amounts of contrast medium entered the duodenum and jejunum(Fig. 2 B).The postoperative recovery of the child was good. Preoperative MRCP and IOC were shown in Figs. 1 and 2.

## Discussion and conclusions

**Fig. 3:**
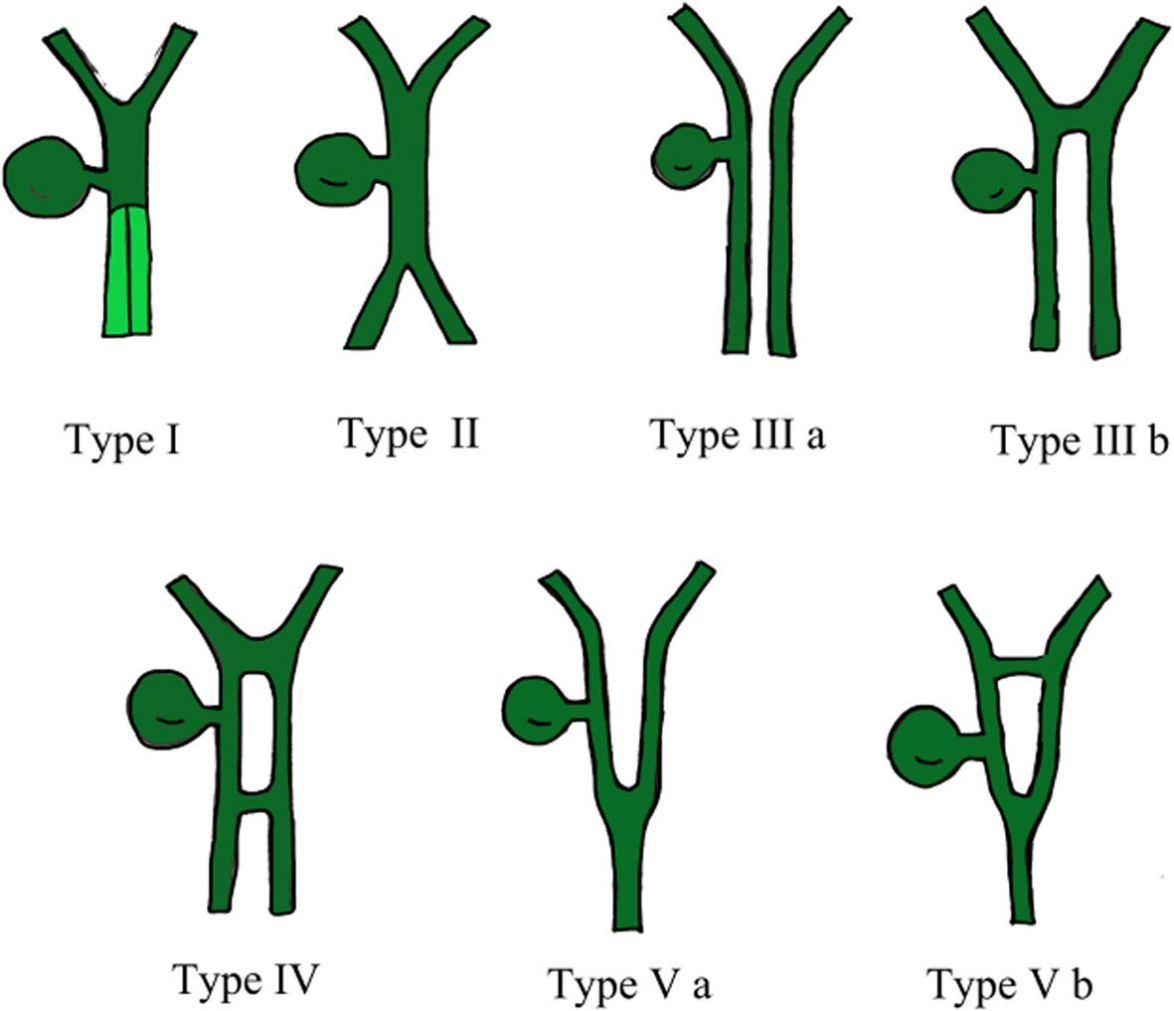
Duplication common bile duct classification proposed by Choi et al. Type I:septum separatting CBD; Type II: CBD bifurcates to drain separately; Type III: double biliary drainage without (**a**) or with (**b**) intrahepatic communicating channels; Type IV: double biliary drainage with extrahepatic communicating channels; Type V: single biliary drainage of double commen bile ducts without (Va) or with (Vb) communicating channels

**Fig. 4:**
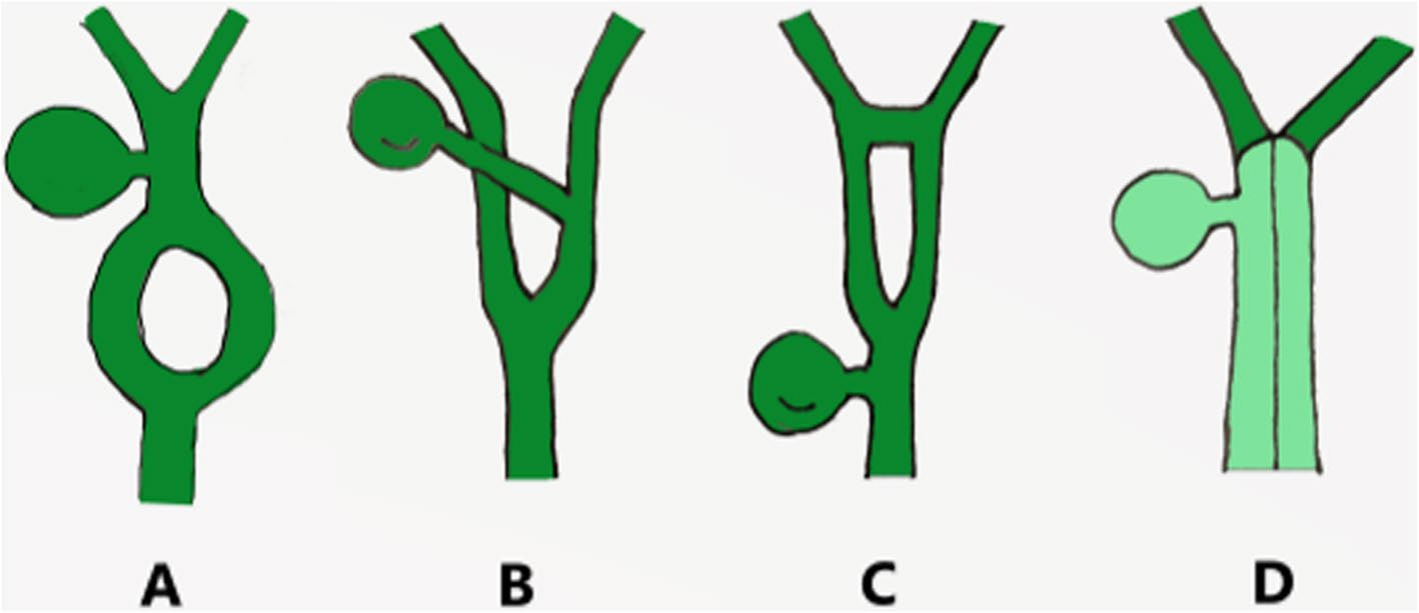
Unclassified reported variants. **A** Variant described by Paraskevas et al. [8]. **B** Variant described by Kosar et al. [9]. **C** Variant described by Nuamah et al. [10, 11]. **D** Variant described in our report

**Fig. 5:**
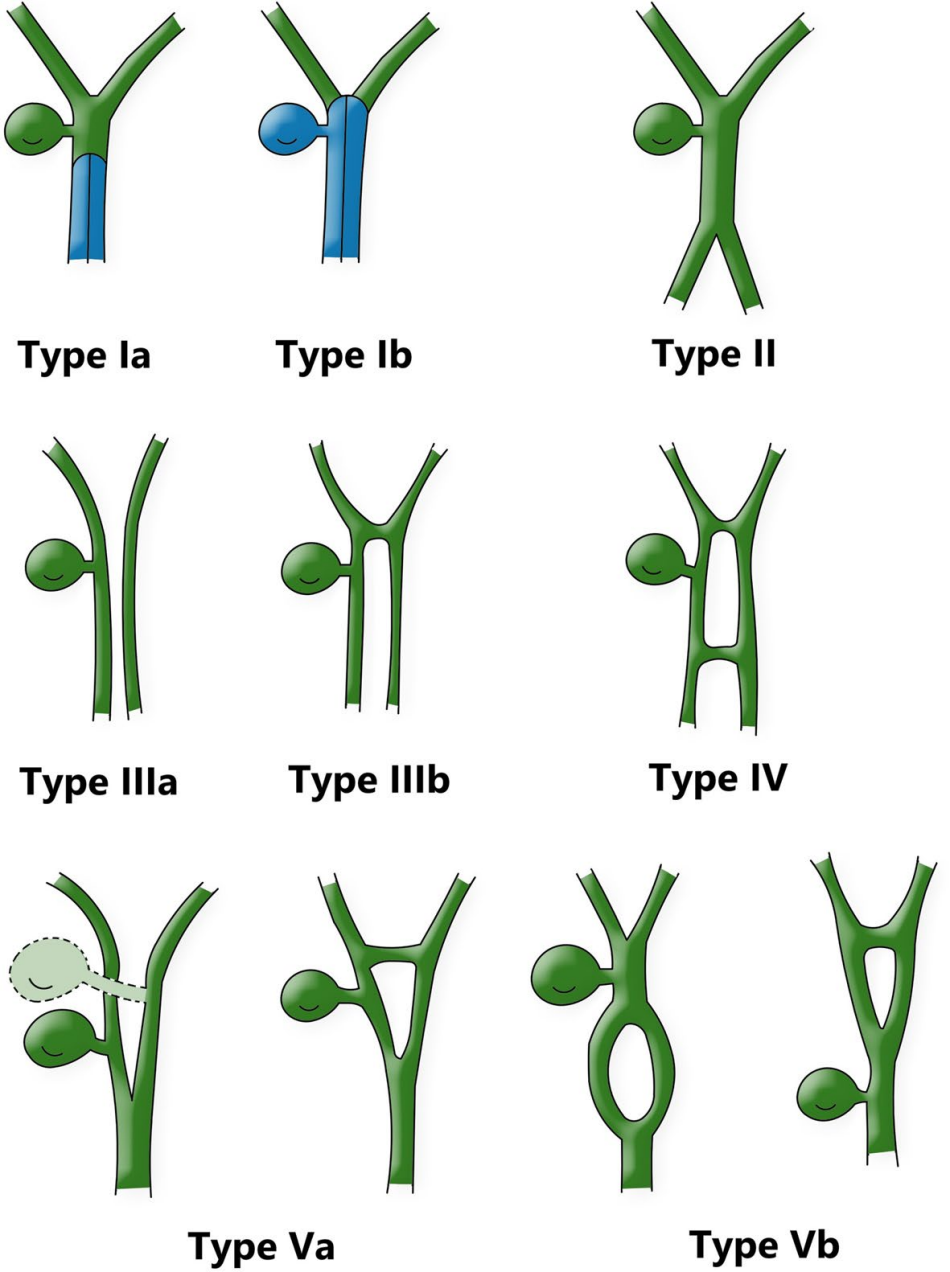
Double common bile duct classification.Modified classification from Choi et al. [7].Type I, Partially (**a**) or completely (**b**) septum within the lumen; Type II, the distal bile duct bifurcates to two independent drainages; Type III, double biliary drainage without any communication (**a**), with intrahepatic communication (**b**); Type IV, double biliary drainage with extrahepatic communicating channels; TypeV, duplicated commen bile ducts join as a single biliary drainage channels,the gallbladder is attached to the repeated biliary ducts or left or right hepatic duct (**a**), The gallbladder is connected to the common bile duct before or after separation (**b**)

Duplication of the common bile duct is an extremely rare condition. The presence of double bile ducts is a normal step in human early embryogenesis. The definite lumen of the bile tree is developed by epithelial proliferation and vacuolization. As the vacuoles coalesce, they will initially create two parallel channels, which will gradually recede to form an isolated common anatomical structure of the common bile duct [4]. Regression failure of the double biliary system is considered to be the mechanism of type I anomaly [5].Chance elongation and early subdivision of the primitive hepatic furrow may be responsible for the other types of DCBD [6].The latest classification system of the DCBD proposed by Choi et al. [7] (2007) based on morphology which did not take into account of the aberrant CBD exits. They described five subtypes involving seven variants (Fig. 3). Since then, three new variants of double common bile duct were reported consecutively, however, none of these were classified into classification system [8–11] (Fig. 4). Our case is a new variant of diaphragmatic common bile duct duplication type I.The reason that why our case was classified as type I was that it was confirmed intraoperatively that there was a septum in common bile duct rather than two separate common bile ducts. The septum of original type I was located in the common bile duct. In our case, the septum in the common bile duct extended to the junction of the left and right hepatic ducts. The above-mentioned variants need a comprehensive classification to encompass these newly discovered variants. Thus, our classification system is as follows (Fig. 5): Type I, Partially (a) or completely (b) septum within the lumen; Type II, the distal bile duct bifurcates to two independent drainages; Type III, double biliary drainage without any communication (a), with intrahepatic communication (b); Type IV, double biliary drainage with extrahepatic communicating channels; TypeV, duplicated commen bile ducts join as a single biliary drainage channel,the gallbladder is attached to the repeated biliary ducts or Left or right hepatic duct (a), the gallbladder is connected to the common bile duct before or after separation(b). Our classification is including all types of common bile duct duplication reported in the present literature. Because of the location of the gallbladder junction and the location of the traffic between the repeated biliary ducts, our classification may contain additional variations not found.

The proportion of type I DCBD is higher in the Chinese population than any other types [12]. DCBD is related to anomalous biliopancreatic junction, congenital choledochal cysts, and biliary atresia and can give rise to complications such as choledocholithiasis, cholangitis, pancreatitis, and malignancies [13]. Its main symptoms include upper abdominal pain, nausea or vomiting, pain in the upper right quadrant, heartburn, fever and jaundice. When clinical manifestations of these hepatobiliary problems occur, the primary imaging approach is usually ultrasound examination in children. Ultrasound examination can clearly show and diagnose choledochal dilatation or biliary tract calculi, but may not show subtle structural abnormalities. In our case, the initial ultrasound examination did not observe repeated common bile duct [14]. The current gold standard for diagnosing biliary tract diseases is endoscopic retrocholangiopancreatography (ERCP), an invasive technique in which contrast agents are injected directly into the gallbladder or bile ducts, which can lead to serious complications, including pancreatitis and duodenal perforation. In addition to ultrasound, MRCP and CT are the most commonly used imaging methods. DCBD was shown by Sang et al. [15] through the minimum density projection using the post-processing technology of multi-slice spiral CT. However, CT cannot be used as a routine preoperative examination for biliary tract diseases in children due to radiation problems. MRCP is a non—invasive examination without injecting contrast agent and without radiation injury, thus is usually the first choice for screening the biliary tract diseases in children requiring surgery. Examination of biliary trees by MRCP may be useful in the diagnosis of other associated congenital anomalies and pathological changes.

In summary, we report a rare case of duplication of the common bile duct combined with congenital biliary dilatation. We complement and adjust the classification of common bile duct duplication which includes all of variations been reported. The primary imaging approach is usually ultrasound. MRCP is usually the first choice for screening duplication of the common bile duct in children requiring surgery.

## Data Availability

The datasets used and/or analyzed during the current study are available from the corresponding author on reasonable request.

## Abbreviations

DCBD: Duplication of the common bile duct
MRCP: Magnetic resonance cholangiopancreatography
IOC: Intraoperative cholangiography
ERCP: Endoscopic retrocholangiopancreatography
